# Incidence, prevalence and care of type 1 diabetes in children and adolescents in Germany: Time trends and regional socioeconomic situation

**DOI:** 10.25646/11439.2

**Published:** 2023-06-14

**Authors:** Maike Buchmann, Oktay Tuncer, Marie Auzanneau, Alexander J. Eckert, Joachim Rosenbauer, Lukas Reitzle, Christin Heidemann, Reinhard W. Holl, Roma Thamm

**Affiliations:** 1 Robert Koch Institute, Berlin, Department of Epidemiology and Health Monitoring; 2 Ulm University, Institute for Epidemiology and Medical Biometry, ZIBMT; 3 German Diabetes Center, Leibniz Center for Diabetes Research at Heinrich Heine University Düsseldorf, Institute for Biometrics and Epidemiology; 4 German Center for Diabetes Research (DZD), München-Neuherberg

**Keywords:** TYPE 1 DIABETES, CARE, COMPLICATIONS, DIABETES SURVEILLANCE, HEALTH MONITORING

## Abstract

**Background:**

Trends over time and possible socio-spatial inequalities in the incidence and care of type 1 diabetes mellitus (T1D) in children and adolescents are important parameters for the planning of target-specific treatment structures.

**Methodology:**

The incidence and prevalence of type 1 diabetes, diabetic ketoacidosis and severe hypoglycaemia as well as the HbA1c value are presented for under 18-year-olds based on data from the nationwide Diabetes Prospective Follow-up Registry (DPV) and the diabetes registry of North Rhine-Westphalia. Indicators were mapped by sex over time between 2014 and 2020, and stratified by sex, age and regional socioeconomic deprivation for 2020.

**Results:**

In 2020, the incidence was 29.2 per 100,000 person-years and the prevalence was 235.5 per 100,000 persons, with the figures being higher in boys than in girls in either case. The median HbA1c value was 7.5%. Ketoacidosis manifested in 3.4% of treated children and adolescents, significantly more often in regions with very high (4.5%) deprivation than in regions with very low deprivation (2.4%). The proportion of severe hypoglycaemia cases was 3.0%. Between 2014 and 2020, the incidence, prevalence and HbA1c levels changed little, while the proportions of ketoacidosis and severe hypoglycaemia decreased.

**Conclusions:**

The decrease in acute complications indicates that type 1 diabetes care has improved. Similar to previous studies, the results suggest an inequality in care by regional socioeconomic situation.

## 1. Introduction

Type 1 diabetes mellitus (T1D) is understood to be an autoimmune disease: an immune system-induced destruction of the insulin-producing cells in the pancreas leading to absolute insulin deficiency [1]. The development of the disease is assumed to be determined by an interplay of genetic and environmental factors [2, 3]. T1D develops more commonly in childhood and adolescence than in adulthood, unlike type 2 diabetes (T2D), which is associated with a relative insulin deficiency [4]. The disease can cause damage to small and large blood vessels and, in due course, to vital organs as well. Long-term complications during the course of disease include cardiovascular disease [5–7], eye and kidney disease [8] and amputations [9]. Given the serious health consequences, T1D can reduce both quality of life [10] and life expectancy [11]. High-quality T1D care, especially during childhood and adolescence, is crucial for achieving optimal metabolic control and health in the later stages of life of the afflicted and is highly relevant in public health.

Approximately 1.5 million people under the age of 20 being afflicted by T1D in 2021 throughout the world [12] make this one of the most common chronic metabolic diseases in childhood and adolescence, and a global increase in the rate of new diagnoses (incidence rate) by 3 to 4% per year has been observed in recent decades, with strong regional differences [13]. Against the background of changing living and environmental conditions, which are discussed as reasons for the increase, the development of the incidence must be monitored reliably and promptly. To be able to make an optimal offer of prevention and care services of the health care system, it is just as important to reliably map the proportion of T1D patients at a certain time (prevalence) as well as the development of the quality of T1D care provided (e.g. long-term blood glucose level, acute complications) and long-term complications (e.g. kidney disease, nerve damage) [14]. One of the aims of the National Diabetes Surveillance established at the Robert Koch Institute (RKI) is to provide, and regularly update, central results on these public health-relevant indicators in childhood and adolescence based on regional and supraregional registry data [15].

The long-term blood glucose value HbA1c (proportion of haemoglobin bound to glucose in total haemoglobin) reflects the average blood glucose level of the previous two to three months and is used to assess the quality of diabetes care. In children and adolescents, the current German guidelines for T1D therapy recommend a value <7.5%, whereas international guidelines recommend <7.0% [16, 17]. In therapy, a balance must be attained between the increased risk of hypoglycaemia upon intensive reduction of glucose levels and the increased risk of secondary diseases in the presence of long-term high blood glucose levels [16, 18].

Diabetic ketoacidosis is one of the common acute complications during the course of this disease. It is a severe metabolic derailment due to insulin deficiency, e.g. during an interruption of therapy or febrile infections. It involves a major increase in the blood sugar level and formation of so-called ketone bodies [18]. This complication can lead to brain oedema (swelling of the brain) in the short term [19] and can be fatal in rare cases. In the long term, it is associated with an unfavourable further course of the diabetes disease [20, 21], kidney damage [22] and cognitive impairment [23]. Age-appropriate diabetes training can have a preventive effect, as can good educational and psychological support for children and adolescents [24].

Hypoglycaemia is an acute complication that is associated with overly low blood glucose levels (low blood sugar) and can become manifest mainly in the context of intensive insulin therapy. The complication manifests when insulin intake is too high relative to the amount of sugar supplied by food or when glucose consumption is increased due to physical activity [18]. Depending on its severity, it can lead to unconsciousness, seizures and, in extreme cases, death; often hospitalisation is required [21, 25]. Hypoglycaemia can be prevented to a large degree by the currently common forms of insulin therapy (conventional and intensive injection therapy, insulin pump therapy), if necessary combined with the use of continuous glucose monitoring (CGM) devices or systems for automated insulin delivery (AID), and training of the patients [26–29]. When hypoglycaemia becomes manifest, hospital admission can be avoided through rapid intake of sugar-containing foods or drinks or through glucagon intake (an antagonist of insulin, e.g. as a nasal spray) [30].

Health inequalities in relation to diabetes have been reported in many studies. For adults in Germany, there continues to be a clear correlation between socioeconomic status (according to educational status) and prevalence of known and undiagnosed diabetes. In contrast, at least for some selected type 2 diabetes care indicators (e.g. HbA1c therapy target), no marked differences between educational groups are evident [31]. Dependence on socioeconomic status has been reported for children and adolescents, internationally as well as in Germany, with regard to the utilisation of medical-technical aids in T1D care and with regard to the HbA1c value [32]. In addition to the fear of incurring costs (despite reimbursement by health insurance funds), lower health literacy and lower self-efficacy of parents are assumed to be some of the possible reasons for socioeconomic differences in treatment, since the utilisation of medical care by children and adolescents is strongly dependent on the parents [33]. For this reason, there is a need to consider regional socioeconomic differences in the incidence, metabolic state and care of diabetes.

The aim of this paper is to present the incidence and prevalence of type 1 diabetes as well as selected indicators of the quality of care (in the following: care indicators; long-term blood glucose value HbA1c, proportion of ketoacidosis and hypoglycaemia) in children and adolescents in Germany for the year 2020, differentiated by sex, age and regional socioeconomic deprivation. In addition, an overview of the development of these indicators over time between 2014 and 2020 is provided.

## 2. Methods

### 2.1 Data sources

The nationwide Diabetes Prospective Follow-up Registry (DPV) was used to estimate nationwide incidences and prevalences of type 1 diabetes in children and adolescents as well as the care indicators. The documentation began in 1995 and is a computer-supported longitudinal recording of treatment-relevant data of diabetes patients that has been continuously developed at Ulm University, Germany. The aim of the DPV initiative, which has more than 400 treatment facilities in Germany, Austria, Luxembourg and Switzerland participating, is to improve the treatment outcomes of people afflicted by diabetes [34, 35].

The analyses of incidence and prevalence over time from 2014 to 2020 are based on the DPV data status of 16 March 2022. The stratified incidence and prevalence analyses for the year 2020 are based on the data status of 03 October 2022. For estimation of the completeness of coverage of the DPV, data from the diabetes registry of the German Diabetes Center (DDZ; North Rhine-Westphalia 0–34 years) were used and, on this basis, coverage-corrected nationwide incidence and prevalence values for under 18-year-olds were calculated [34, 36]. The care indicator results for the trend over time and the stratified analysis for the year 2020 are based on the data status of 28 November 2022 with the data from the DPV software made anonymous.

In the incidence and prevalence estimates, registered persons were included if they were under 18 years of age at the time of diagnosis of the disease or at the end of the calendar year. For the calculations on the care indicators for 2020, 24,978 children and adolescents with T1D from the DPV Registry ranging in age from half a year to under 18 years, who had been diagnosed for at least three months and had at least one documented examination in the treatment year 2020, were included in the analysis.

#### Variables

Registry-based estimated incidences and prevalences served as indicators for the descriptive analysis.

The care indicator HbA1c value was presented as median with interquartile range (IQR). In each case, all HbA1c readings of the respective year were used and aggregated into the median for each person. The values were standardised according to the reference range of the Diabetes Control and Complications Trial [37]. Further care indicators considered herein were hospital-treated diabetic ketoacidosis in the course of the disease, severe hypoglycaemia (self-reported hypoglycaemia with the person dependent on outside help), severe hypoglycaemia associated with unconsciousness and severe hypoglycaemia associated with subsequent hospitalisation, as percentages relative to the group of children and adolescents with type 1 diabetes.

**Figure 1:**
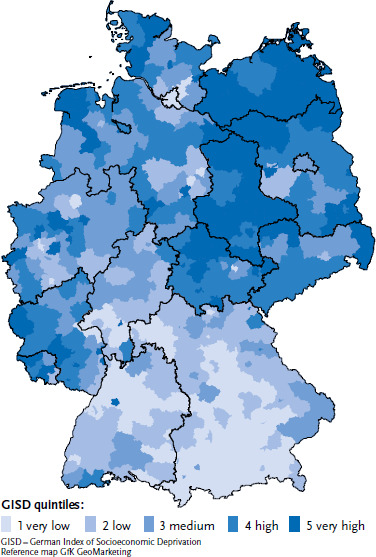
Distribution of regional socioeconomic deprivation at district level (as of 31 December 2019) Source: Federal Agency for Cartography and Geodesy (2022); GISD Release 2022 v0.2

The German Index of Socioeconomic Deprivation (GISD) was used to examine the selected indicators from the perspective of health inequality. The GISD maps socioeco nomic differences on a spatial level [38]. Based on the ‘Indicators and Maps of Spatial and Urban Development’ (INKAR) database of the Federal Institute for Research on Building, Urban Affairs and Spatial Development, an index of the socioeconomic deprivation was formed from nine indicators of three dimensions, i.e. education, occupation and income, that were available for different regional levels. The index enables comparisons to be made of the socioeconomic deprivation for the 401 districts and independent cities in Germany (as of 31 December 2019) [38]. The districts are sub-divided into quintiles and a distinction is made between districts with ‘very low’ (GISD quintile 1), ‘low’ (GISD quintile 2), ‘medium’ (GISD quintile 3), ‘high’ (GISD quintile 4) and ‘very high’ (GISD quintile 5) socio-economic deprivation. Figure 1 shows the distribution of GISD quintiles at the district level in Germany.

Regions with very high socioeconomic deprivation (darkest colouring) are mainly located in the new federal states and in parts of Schleswig-Holstein, North Rhine-Westphalia, Rhineland-Palatinate and Saarland. Throughout Baden-Württemberg and Bavaria, there are some districts with very low socioeconomic deprivation.

In the present analysis, the GISD quintiles were assigned to individuals on the basis of their place of residence. For the care indicators, the postcode of the person’s place of residence was used. For incidence and prevalence, the district code of the place of residence was also used.

### 2.2 Statistical methods

For incidence and prevalence, diabetes cases per 100,000 person-years or persons were calculated using population figures from the Federal Statistical Office for each year between 2014 and 2020 as an update of the 2011 census [39]. Due to the availability of three data sources for North Rhine-Westphalia, it was possible to estimate the completeness of coverage of the individual sources including the DPV Registry for type 1 diabetes in the age group 0–17 years using capture-recapture methods. Assuming the estimated completeness of coverage of the DPV Registry in North Rhine-Westphalia to be a good estimate of the nationwide completeness, which is quite plausible, the nationwide DPV data were then corrected for undercoverage and coverage-corrected estimates of incidence and prevalence were calculated for Germany.

Completeness of coverage was estimated according to a log-linear model. For this purpose, log-linear models were fitted to the data taking into account overdispersion, and the model that best described the registry data was selected according to the AICC criterion [40–42]. Assuming the cases to fit a Poisson distribution, incidences and prevalences were estimated with 95% confidence intervals using the person-year method and were directly sex- and/or age-standardised, and trends over time of incidence and prevalence were calculated using Poisson regression analyses [43]. Due to the values being low, the prevalence is not given as a percentage, as is common practice, but as diabetes cases per 100,000 persons.

For the year 2020, the indicators were analysed differentiated by sex, age and regional socioeconomic deprivation. Five age groups were taken into consideration: under 3 years, 3 to 6 years, 7 to 10 years, 11 to 13 years and 14 to 17 years of age. As the number of cases for the care indicators in the lowest two age groups was too small, these were combined into an under-7-years age group for severe hypoglycaemia, diabetic ketoacidosis and HbA1c value. For the estimated T1D incidence and prevalence values by GISD quintiles, direct age and sex standardisation was performed based on the age groups listed and with equal weighting of the sexes. In addition, the association between incidence, prevalence and regional socioeco nomic deprivation was investigated in a Poisson model, adjusted for geographical location (North/Central/South), age and sex.

For the time trends from 2014 to 2020, the estimators for incidence, prevalence and care indicators are presented overall and differentiated by sex.

Statistically significant differences were assumed to exist if either the 95% confidence intervals do not overlap or if the corresponding p-values from Chi-square test of independence or Wilcoxon tests were ≤0.05. Statistical analyses were performed using the SAS statistical package, version 9.4 (SAS Institute, Cary, NC, USA); maps were produced using the free open source software QGIS 3.26.2 [44].

## 3. Results

### 3.1 Incidence and prevalence of type 1 diabetes in children and adolescents in Germany

Table 1 shows the coverage-corrected nationwide incidence and prevalence estimates for the year 2020 overall, by sex and age, and age- and sex-standardised by regional socioeconomic deprivation. The overall estimated incidence of T1D is 29.2 per 100,000 person-years, which corresponds to an estimated number of 4,044 new diagnoses in 2020 (girls: 1,784; boys: 2,260). The overall T1D incidence is higher for boys than for girls (31.9 vs. 26.5 per 100,000 person-years, respectively), with statistically significant differences in incidence by sex being evident in the age groups of 11 to 13 years and 14 to 17 years. The incidence is highest in girls in the age groups of 7 to 10 and 11 to 13 years (37.1 and 35.9 per 100,000 person-years, respectively), and in boys in the age group of 11 to 13 years (48.8 per 100,000 person-years). Differentiating by regional socioeconomic deprivation, a higher incidence is seen in districts with very high deprivation (31.9 per 100,000 person-years) than in districts with very low deprivation (27.3 per 100,000 person-years).

In 2020, the estimated nationwide prevalence of T1D in children and adolescents was 235.5 per 100,000 persons (Table 1). This corresponds to an estimated number of 32,230 persons (girls: 15,239; boys: 16,991). The T1D prevalence is higher in boys than in girls (241.4 vs. 229.5 per 100,000 persons, respectively), with differences by sex being statistically significant in the age groups of under-3-year-olds and 14 to 17-year-olds. As expected, there is a dependence of T1D prevalence on age, with higher prevalences being observed in older age groups. With regard to regional deprivation, regions with very low and low socioeconomic deprivation show statistically significantly lower prevalences of T1D than regions with medium to very high deprivation (Table 1).

**Figure 2:**
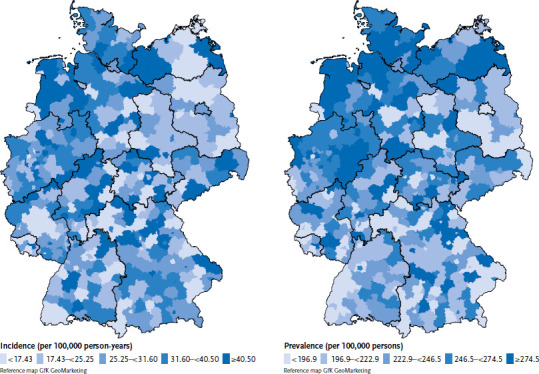
Incidence (per 100,000 person-years, n=1,784 girls, n=2,260 boys) and prevalence (per 100,000 persons, n=15,239 girls, n=16,991 boys) of type 1 diabetes in children and adolescents in 2020 at district level Source: DPV Registry; data status: 03 October 2022

Looking at the geographical situation, no consistent pattern emerges in the distribution of T1D incidence and prevalence in 2020 at the district level (Figure 2). Clusters of low-incidence districts tend to be seen in Saxony-Anhalt and Brandenburg, whereas a clustering of high-incidence districts tends to be seen in the Northwest. Similarly, there is marked clustering of high prevalences in the Northwest. When viewed in conjunction with Figure 1, it is evident that the distribution patterns of incidence, prevalence and regional socioeconomic deprivation at district level are not congruent. The association between incidence and sociospatial deprivation that is evident from Table 1 on the basis of the stratified analysis is no longer seen in a Poisson model after adjustment for geographical location (North/Central/South) (Annex Table 1).

**Figure 3:**
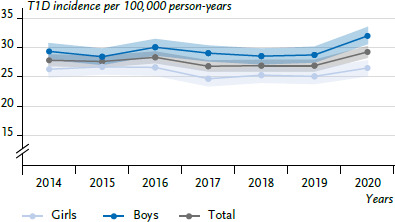
Incidence of type 1 diabetes in children and adolescents over time (2014–2020) by sex (n=11,819 girls, n=14,261 boys) Source: DPV Registry; data status: 16 March 2023

Between 2014 and 2020, there is no clear overall trend with regard to the incidences (Figure 3). For 2020, there is a statistically significant increase in the incidence, both overall and in boys, compared to 2019 (overall 2019: 27.0, 95% CI: 26.1–27.9, 2020: 29.3, 95% CI: 28.4–30.2; boys 2019: 28.8, 95% CI: 27.6–30.1, 2020: 32.0, 95% CI: 30.7–33.4). In the observation period from 2014 to 2020, new diagnoses are estimated to a total of 26,080 (girls: 11,819, boys: 14,261; average 3,725 per year) and the average incidence, standardised for age and sex, is 27.7 cases of diabetes per 100,000 person-years (girls: 26.0; boys: 29.5). In each of the years considered except 2015, the incidence is signifiicantly higher in boys than in girls. The incidence per 100,000 person-years for girls is lowest in 2017 (24.8) and highest in 2015 (26.8); for boys, it is lowest in 2015 (28.5) and highest in 2020 (32.0).

**Figure 4:**
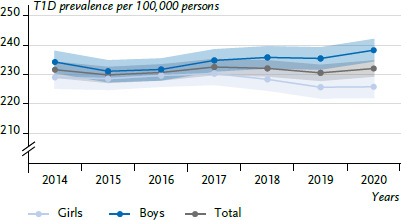
Prevalence of type 1 diabetes in children and adolescents over time (2014–2020) by sex (n=104,979 girls, n=114,736 boys) Source: DPV Registry; data status: 16 March 2023

Over time, the nationwide T1D prevalence in individuals under 18 years of age shows some fluctuation from 231.8 in 2014 to 232.2 per 100,000 persons in 2020 (Figure 4; annual change, girls: 0.2% (-0.2–0.7, p=0.325); boys: 0.4% (-0.2–1.1, p=0.221)). On average, the prevalence is 231.5 per 100,000 persons. Starting from 2018, significantly higher prevalences are observed for boys than for girls.

### 3.2 Quality of care

In 2020, statistically significantly higher HbA1c values were measured in girls than in boys (7.6%; IQR: 6.9–8.4 versus 7.5%; IQR: 6.8–8.3, p<0.001). In increasingly older age groups, the median increased slightly from 7.3% in the under-7-years age group to 7.8% in the 14 to 17-years age group.

The median measured HbA1c value in regions with very low socioeconomic deprivation was 7.4% (IQR: 6.8–8.1) and was statistically significantly higher in regions with very high socioeconomic deprivation, where it was 7.7% (IQR: 7.0–8.6, p<0.001). Overall, there was a gradient in median HbA1c values from regions with low socioeconomic deprivation to regions with high deprivation (Table 2).

The median HbA1c values did not change between 2014 (Girls: 7,5% (IQR: 6,8–8,4); Boys: 7,5% (IQR: 6,8–8,3)) and 2020 (Girls: 7,6% (IQR: 6,9–8,4); Boys: 7,5% (IQR: 6,8–8,3)), apart from minor fluctuations between 2016 and 2018.

A total of 3.4% of the children and adolescents with T1D were hospitalised for diabetic ketoacidosis at least once in 2020. Overall, the proportion of girls experiencing diabetic ketoacidosis at least once was statistically significantly higher (3.8%; 95% CI: 3.4–4.1) as compared to boys (3.2%; 95% CI: 2.9–3.5, p=0.03). In children and adolescents from the age of 11, the proportions of diabetic ketoacidosis were significantly higher (in 11 to 13-year-olds: 3.6%; 95% CI: 3.2–4.0; in 14 to 17-year-olds: 4.0%; 95% CI: 3.7–4.4) than in children and adolescents under 11 years of age (under 7 years of age: 2.6%; 95% CI: 2.1–3.1; in 7 to 10-year-olds: 2.3%; 95% CI: 2.0–2.7) (Table 3).

The proportion of all children and adolescents with T1D who had diabetic ketoacidosis in 2020 was statistically significantly lower in regions with the lowest deprivation quintile (2.4%; 95% CI: 2.1–2.7) than in the other deprivation quintiles.

**Figure 5:**
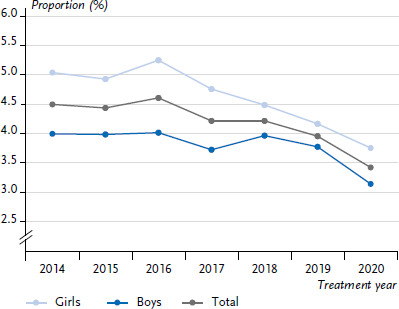
Proportion of children and adolescents with type 1 diabetes over time (2014–2020) experiencing diabetic ketoacidosis at least once (n=79,462 girls, n=87,917 boys) Source: DPV Registry; data status: 29.11.2022

Looking at the trend over time, the proportion of children and adolescents experiencing diabetic ketoacidosis has decreased between 2014 and 2020 (Figure 5). It decreased from 4.5% in 2014 to 3.4% in 2020. The proportions decreased more strongly in girls than in boys (girls: 2014: 5.1%, 2020: 3.8%; boys: 2014: 4.0%, 2020: 3.2%).

Severe hypoglycaemia manifested in 3.0% of children and adolescents with T1D in 2020, with no statistically significant differences by sex and age (Table 3). In 2020, 0.7% of children and adolescents with T1D experienced severe hypoglycaemia with unconsciousness at least once and 0.5% had severe hypoglycaemia with hospitalisation at least once. There were no statistically significant differences by sex or age for these two indicators either (Annex, Table 2).

**Figure 6:**
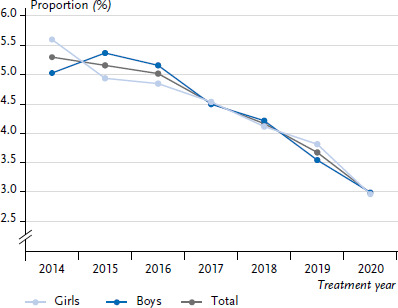
Proportion of children and adolescents with type 1 diabetes over time (2014–2020) experiencing severe hypoglycaemia at least once (n=79,462 girls, n=87,917 boys) Source: DPV Registry; data status: 29.11.2022

The proportion of children and adolescents experiencing severe hypoglycaemia at least once in a year shows a strong decrease over the observation period from 2014 to 2020 (Figure 6). In 2014, the proportion was 5.3% (95% CI: 5.0–5.6); in 2020 it had dropped to 3.0% (95% CI: 2.8–3.2). A strong decrease is noted both in boys (2014: 5,0%; 2020: 3.0%) and girls (2014: 5,6%; 2020: 3.0%).

Both the proportions of severe hypoglycaemia with unconsciousness and severe hypoglycaemia with hospitalisation showed a steady annual decrease between 2014 and 2020 (Annex, Figure 1). In 2014 and 2020, the proportion of treated children and adolescents with type 1 diabetes experiencing severe hypoglycaemia with loss of consciousness was 1.7% (95% CI: 1.6–1.9) and 0.7% (95% CI: 0.6–0.8), respectively. In 2014, 1.2% (95% CI: 1.0–1.3) experienced severe hypoglycaemia and were admitted to hospital; in 2020, this proportion was 0.5% (95% CI: 0.4–0.6).

## 4. Discussion

### 4.1 Incidence and prevalence of T1D in children and adolescents

#### Interpretation of the results and comparison to other studies

Globally, the incidence of type 1 diabetes in children and adolescents is estimated to have increased by 3 to 4% each year on average over the past decades [13, 45]. This estimate is consistent with the annual 3.4% increase in incidence in children and adolescents up to 14 years of age in Germany, which was calculated on the basis of registry data from the period of 1999 to 2008 [36]. In contrast, as described here, no significant change in the incidence of type 1 diabetes in under-18-year-olds in Germany is detectable between 2014 and 2020. A levelling off of the increase or a decrease in incidence in recent years has also been noted in other countries, for example in Sweden [46] and Finland [47].

In a global comparison, the country-specific incidence rates and trends over time vary strongly [48–50]. Indeed, for the 0–14 years age group, they range from less than 5 per 100,000 person-years (e.g. Japan: 2.2) to more than 30 per 100,000 person-years (e.g. Finland: 52.2) [49, 50]. Regional differences in the development of the incidence over time are evident within Germany as well [14].

The overall T1D prevalence in children and adolescents is almost unchanged between 2014 and 2020. Prevalence estimates based on data for children and adolescents aged 0–19 years for the period from 2002 to 2020 in North Rhine-Westphalia are indicative of a similar prevalence of 247.1 per 100,000 persons (95% CI: 240.3–253.9), with an annual increase of 2.9% (95% CI: 2.7–3.1) [51]. Data that is also based on the nationwide DPV Registry show that the previous significant increase in T1D prevalence levelled off between 2002 and 2020 (increase between 2002 and 2008: 6.3%; 2008–2014: 3.1%; 2014–2020: 0.5%), and that the prevalence increased only in the age group of 15 to 19 years of age since 2014 [52]. For 2020, it was observed that preva lence increased by age group, as expected. This is consistent with findings from North Rhine-Westphalia from 2010 [14].

For 2020, higher T1D incidence values are evident in districts with very high deprivation as compared to districts with very low deprivation. However, after adjustment for geographical location (North/Central/South), there is no longer any evidence of an association to exist between regional socioeconomic deprivation according to the GISD at the district level and the T1D incidence in children and adolescents in Germany. Data on new diagnoses in persons under 20 year of age for the period between 2007 and 2014 in North Rhine-Westphalia show a higher incidence (relative to environmental factors and employment rate) in less deprived communities as well as in more remote regions. The association between incidence and degree of urbanisation is stronger than the association between incidence and socioeconomic deprivation [53]. This is consistent with data on the incidence of type 1 diabetes in children and adolescents between 0 and 14 years of age in North Rhine-Westphalia for the period from 1996 to 2000 [54].

The underlying data demonstrated a higher T1D prevalence in districts with higher socioeconomic deprivation. Data from England and Wales show only insignificant differences in T1D prevalence by socioeconomic deprivation, at best being somewhat indicative of a higher proportion of children with T1D in regions of highest deprivation [55]. While there may be explanations for an increased T2D prevalence in more deprived regions (e.g. limited access to exercise opportunities, higher proportion of obesity) [55], the association with regard to T1D is unclear and not unambiguous.

Interpreting the regional socioeconomic contextual differences in incidence and prevalence, a possible influence of other factors such as environmental factors exhibiting geographical differences should be taken into consideration. By the eye test, there is no significant association between incidences or prevalences and GISD (Figure 2). Overall, international ecological analyses of the incidence of type 1 diabetes yielded inconsistent results, so that there is no clear evidence yet on the influence of spatial socioeconomic or climatic factors on the incidence [48]. The regional differences in the incidence of type 1 diabetes and the changes over time indicate that the disease process is multifactorial [48]. The extent to which genetic and/or environmental factors affect the risk of disease is still the subject of research. Environmental factors that are closely related to the degree of urbanisation and the socioeconomic status are discussed as being relevant for the disease, though the evidence is inconsistent [48]. There is evidence that early childhood exposure to pathogens, more common in high population density settings, may be protective against the development of type 1 diabetes in those with a genetic predisposition. Accordingly, in some countries, low incidence rates were linked to high population density, whereas in other countries high incidence rates were detected in urban areas. Different definitions of rural and urban areas and differences in socioeconomic deprivation are being discussed as possible causes of these contradictory observations [48]. Furthermore, there are indications for a association to exist between the incidence of type 1 diabetes and climatic conditions [56].

The increase in incidence in 2020 compared to the previous year, both overall and in boys, should also be evaluated against the background of the COVID-19 pandemic. An analysis from 2022 showed an unexpected increase in the incidence of T1D in the first 1.5 years of the pandemic [57]. For example, between 1 January 2020 and 30 June 2021, the incidence was 24.4 per 100,000 person-years (95% CI: 23.6–25.2) exceeding the expected incidence of 21.2 (95% CI: 20.5–21.9), and resulted in an ‘incidence rate ratio’ (IRR) of 1.15 (95% CI: 1.10–1.20), meaning that the incidence was about 15% higher than expected based on the long-term trend. There were no differences between boys and girls. Stratified by age groups, there was an increased IRR for children under 6 years of age (1.23; 95% CI: 1.13–1.33) and between 6 and 11 years of age (1.18; 95% CI: 1.11–1.26), but not in children and adolescents older than these ages [57]. A meta-analysis of 24 international studies provided evidence of a statistically significant association to exist between the COVID-19 pandemic and a worldwide increase in T1D incidence in children and adolescents [58]. Overall, the state of research on the development of T1D incidence is heterogeneous [59]. Further studies are needed to investigate the causes of the increase in T1D incidence. On the one hand, SARS-CoV-2 infection might possibly promote the development of T1D directly. On the other hand, the measures taken to contain the COVID-19 pandemic and the associated stress might have had an indirect influence on the development of the disease [60].

#### Conclusions related to Public Health

The prevalence of T1D is a crucial measure for planning the care to be provided. According to the results, at least an unchanged need for care of children and adolescents with type 1 diabetes must be expected in the upcoming years [14]. The increase in incidence during the COVID-19 pandemic should be kept in mind and should be investigated further [60]. According to the Diabetes Atlas of the International Diabetes Federation, Germany is among the ten countries with the highest incidence of cases and the highest estimated prevalence of type 1 diabetes [50, 61].

### 4.2 Quality of care indicators in children and adolescents with T1D

#### Interpretation of the results and comparison to other studies

The HbA1c values are constant over time. Since the median HbA1c value of 7.5% is just above the recommended value of the German guidelines of <7.5% [16] and above the recommended value of the international guidelines of <7.0% [17], it can be assumed that there is a need for improvement. Consistent with our study, other studies noted significant differences by sex in indicators such as metabolic control and diabetic ketoacidosis over time, and report higher HbA1c values for girls than for boys [62].

For the care indicators considered herein, a positive development is seen over the observation period from 2014 to 2020. The incidence of acute complications in children and adolescents with T1D have decreased during this period, both in girls and in boys.

The evident decrease of diabetic ketoacidoses is mainly due to a strong decrease in girls. Nevertheless, girls were more frequently afflicted by diabetic ketoacidosis than boys in 2020. The higher risk of diabetic ketoacidosis in the older age groups reported in the literature [63] was also observed for the year 2020 in this study. Diabetic ketoacidosis manifested more frequently in children and adolescents with T1D in regions of higher socioeconomic deprivation than in regions of lower socioeconomic deprivation. This observation is consistent with results from other studies [64, 65].

The observed decrease in the proportions of severe hypoglycaemia over the years is consistent with the literature [66], and is quite pronounced in both boys and girls. With regard to the regional socioeconomic context, no clear associations were detected. Only in boys with T1D do the descriptive analyses show significantly more severe hypoglycaemia cases in regions with the lowest socioeconomic deprivation as compared to the other regions. A study using data from 2015 and 2016 [64] reported the incidence of severe hypoglycaemia to gradually decrease from regions with the lowest deprivation to regions with the highest deprivation.

Similarly, between 2014 and 2020, there was a decrease in the proportion of children and adolescents with T1D who experienced hypoglycaemia with unconsciousness or hypoglycaemia with hospitalisation in the respective year of treatment.

#### Conclusions related to Public Health

Looking at the trend over time from 2014 to 2020, it was evident that, for acute complications, the proportions of girls and boys have become equal and significant differences between girls and boys exist only for diabetic ketoacidosis in 2020. Although treatment choices were not a subject of the analysis, one possible explanation for the convergence of the difference by sex is that the use of insulin pumps has become more widespread in girls than in boys, so that insulin pump therapy is now used more commonly in girls than in boys [33, 62].

The prevention of severe hypoglycaemic episodes in particular can be seen as a quality indicator of good glycaemic control and may point to increasing recipient-appropriate training [26]. Hypoglycaemia can have secondary complications such as cardiovascular events and dementia or accidents and fall-related fractures and even death [67]. In recent years, medical-technical aids such as continuous glucose monitoring and insulin pumps, which facilitate the dosing adjustments in type 1 diabetes therapy, have become widespread among children and adolescents in Germany [68]. The risk of hypoglycaemia while on insulin pump therapy is lower than on injection therapy, and on average a better metabolic control is achieved [69, 70]. A total of 93% of all children with T1D under five years of age in 2020 used an insulin pump [69]. Furthermore, an improvement in blood glucose control and a lower average HbA1c value are evident to result from the use of continuous glucose monitoring [71]. In addition, the risk of hypoglycaemia can be reduced even more through a more widespread use of AID systems, which allow for automated cut-off of insulin delivery if hypoglycaemia manifests or before impending hypoglycaemia [30, 72]. In this context, the effects of the spread of medical-technical aids and the correlation with the regional socioeconomic context in children and adolescents should be further investigated in the future.

The HbA1c value is increasingly supplemented by an assessment of the time in target range (time in range, TIR) or time in the hypoglycaemic range (time below range, TbR) acquired from glucose profiles. This also allows short- to medium-term glucose control to be analysed and, aside from the HbA1c value, may possibly be relevant for therapy decisions in the provision of care in the future [73].

Overall, the results indicate that the type 1 diabetes care provided in Germany has improved. However, as both indicators, HbA1c and proportion of diabetic ketoacidosis, show, there continues to be an inequality in the care provided to children and adolescents depending on the regional socioeconomic context, which has been noted previously in other studies [32, 64]. There is evidence indicating that, especially in regions with higher socioeconomic deprivation, it makes sense to involve individuals from other settings (caregivers and peers in daycare centres, schools and leisure time) in the treatment and prevention of complications by means of pertinent training. It is also important to take into account the health literacy and the age of the target group during diabetes trainings [24]. It should be noted here that some patients postponed contact with medical care during the pandemic, so that their follow-up data for 2020 could not be recorded. In order to investigate in more detail the effects of the pandemic on the quality of care provided to children and adolescents with T1D, further analyses focussing on the pandemic period are needed [74, 75]. Effects of the pandemic may also occur with a delay. For this reason, it seems important to track relevant indicators for the years 2021, 2022 and beyond, as younger people were infected predominantly in the later waves of the pandemic.

Furthermore, standardised documentation of type 1 diabetes in children and adolescents, such as the DPV Registry, is important for recurrent and longitudinal analyses (www.d-p-v.eu [35]).

### 4.3 Strengths and limitations

This article summarises data from ongoing diabetes registries in Germany on T1D incidence, prevalence and care, and combines the results with information on GISD to provide a comprehensive overview of trends over time and current differences by regional socioeconomic situation. The nationwide DPV data on incidence and prevalence exhibit a high degree of coverage (completeness), which was estimated using capture-recapture methods based on the data for North Rhine-Westphalia from the DDZ Diabetes Register. Data collection in North Rhine-Westphalia has been conducted for more than 20 years, mainly using the same methodology [36]. The estimates of incidence and prevalence were corrected for the low degree of undercoverage. Nevertheless, it should be noted that the data quality depends on the documentation of the clinics and practices, which voluntarily participate in the registries. The estimates of the incidence and prevalence of T1D and the corresponding trends are based on model analyses and should therefore be seen as approximations. With the multidimensional index of regional socioeconomic deprivation used, an ecological correlation of the indicators considered was conducted at the small-scale regional level. The index can therefore not replace a measurement of socioeconomic status at the individual level.

### 4.4 Conclusion

The incidence and prevalence of type 1 diabetes in children and adolescents have changed only marginally in the period from 2014 to 2020. There are some differences evident upon differentiation by regional socioeconomic deprivation. The association between the regional socioeconomic deprivation and the incidence is not clear and should be the subject of further research, as should the influence of genetic and environmental factors and of the COVID-19 pandemic. In this context, it would be of interest to look at the age distribution of incidence and prevalence values with respect to regional socioeconomic deprivation.

With regard to the long-term blood glucose value HbA1c, no change over time was observed. It should be analysed which measures and interventions might contribute to a long-term reduction of the HbA1c values and thus to improved diabetes care for children and adolescents.

Regarding the acute complications considered, there is an improvement in the trend over time as well as a decrease of the differences between boys and girls. This indicates that the widespread use of medical-technical aids in the care provided to children and adolescents with type 1 diabetes has contributed to a reduction of the complications. Furthermore, a higher proportion of children and adolescents with type 1 diabetes experiencing diabetic ketoacidosis at least once is detected in regions with higher socioeconomic deprivation. It can safely be assumed that, especially in these socioeconomically deprived areas, the care provided for this disease and the education about risk factors and causes of diabetic ketoacidosis can be improved.

The data on the T1D incidence and care in children and adolescents in Germany demonstrate the importance of the registries and of the integration of the registry data into the diabetes surveillance system at the RKI. The aim of diabetes surveillance is a regular indicator-based diabetes reporting to provide timely and action-oriented information in the realms of health policy, health research, health care and public health practice.

**Corrigendum**

In the original version of the article, the source of the GISD data in Figure 1, Table 1, Table 2 and Table 3, Annex Table 1 and Annex Table 2 (‘GISD Release 2022 v0.2’) and information on the availability of the GISD dataset were missing. Reference no. 38 did not cite the most recent paper to the topic.

We added the data source for Figure 1 and the tables, updated the literature, and also included a note on GISD data in the ‘Data availability’ section.

## Key statement

- Approximately 4,000 children and adolescents had a new type 1 diabetes diagnosis in 2020, with the prevalence being approximately 235.5 per 100,000 persons.
- The median long-term blood glucose level HbA1c in children and adolescents with type 1 diabetes in 2020 was 7.5%, while the German guidelines recommend a value <7.5%.
- At least one incident of ketoacidosis requiring hospitalisation occurred in 3.4% of treated children and adolescents with type 1 diabetes, more common in regions of very high socioeconomic deprivation.
- The proportion of severe hypoglycaemia in 0–17 year-olds with type 1 diabetes was 3.0%.
- Between 2014 and 2020, the incidence, prevalence and HbA1c level changed little, while acute complications like ketoacidosis and severe hypoglycaemia decreased significantly.

## Figures and Tables

**Table 1:** Incidence and prevalence of type 1 diabetes in children and adolescents in 2020 by sex, age group and regional socioeconomic deprivation Source: DPV Registry, data status: 03 October 2022; GISD Release 2022 v0.2; own calculations

	Incidence (per 100,000 person-years) (n=1,784 girls, n=2,260 boys)						Prevalence (per 100,000 persons) (n=15,239 girls, n=16,991 boys)					
	Girls		Boys		Total		Girls		Boys		Total	
	(95% CI)		(95% CI)		(95% CI)		(95% CI)		(95% CI)		(95% CI)	
**Age (years)** ^1^												
<3	15.0	(12.9–17.4)	17.0	(14.8–19.5)	16.0	(14.5–17.7)	12.8	(10.8–15.0)	18.8	(16.5–21.4)	15.9	(14.3–17.6)
3–6	27.0	(24.5–29.7)	26.6	(24.2–29.3)	26.8	(25.0–28.7)	88.8	(84.1–93.6)	93.3	(88.7–98.0)	91.1	(87.8–94.4)
7–10	37.1	(34.1–40.4)	39.1	(36.0–42.3)	38.1	(36.0–40.4)	225.2	(217.6–233.0)	217.7	(210.4–225.2)	221.3	(216.0–226.7)
11–13	35.9	(32.4–39.6)	48.8	(44.9–53.0)	42.6	(39.9–45.3)	357.4	(346.4–368.8)	362.8	(351.9–373.9)	360.2	(352.4–368.1)
14–17	17.1	(15.1–19.4)	28.3	(25.7–31.1)	22.9	(21.2–24.7)	441.1	(430.4–452.0)	489.3	(478.3–500.4)	465.9	(458.2–473.6)
**Regional socioeconomic deprivation^2^**												
Very low	24.2	(21.9–26.6)	30.3	(27.7–32.9)	27.3	(25.5–29.0)	213.8	(206.7–220.9)	233.0	(225.8–240.3)	223.4	(218.3–228.5)
Low	28.0	(25.2–30.7)	29.5	(26.8–32.3)	28.8	(26.8–30.7)	217.7	(210.1–225.3)	221.4	(213.9–228.9)	219.5	(214.2–224.9)
Medium	27.6	(25.0–30.3)	33.9	(31.1–36.8)	30.8	(28.8–32.7)	234.7	(227.0–242.5)	246.0	(238.3–253.7)	240.4	(234.9–245.8)
High	25.9	(23.0–28.9)	30.6	(27.4–33.7)	28.2	(26.1–30.4)	246.4	(237.3–255.5)	251.1	(242.1–260.0)	248.7	(242.4–255.1)
Very high	27.4	(24.1–30.7)	36.5	(32.8–40.2)	31.9	(29.4–34.4)	245.4	(235.4–255.3)	267.0	(256.9–277.0)	256.2	(249.1–263.3)
**Total**	**26.5**	**(25.3–27.8)**	**31.9**	**(30.6–33.2)**	**29.2**	**(28.3–30.1)**	**229.5**	**(225.8–233.1)**	**241.4**	**(237.8–245.1)**	**235.5**	**(232.9–238.0)**

^1^ coverage-corrected

^2^ coverage-corrected and standardised for age and sex

**Table 2:** Median HbA1c value in children and adolescents with type 1 diabetes in 2020 by sex, age group and regional socioeconomic deprivation Source: DPV Registry, data status: 15 December 2022; GISD Release 2022 v0.2; own calculations

	Girls		Boys		Total	
	M (%)	(IQR)	M (%)	(IQR)	M (%)	(IQR)
**Age (years)**						
<7	7.3	(6.7–7.9)	7.3	(6.7–7.9)	7.3	(6.7–7.9)
7–10	7.3	(6.7–7.9)	7.2	(6.7–7.9)	7.3	(6.7–7.9)
11–13	7.6	(6.9–8.4)	7.5	(6.8–8.3)	7.5	(6.9–8.3)
14–17	7.8	(7.1–8.7)	7.7	(7.0–8.7)	7.8	(7.0–8.7)
**Regional socioeconomic deprivation**						
Very low	7.4	(6.8–8.1)	7.4	(6.7–8.1)	7.4	(6.8–8.1)
Low	7.5	(6.9–8.2)	7.5	(6.8–8.2)	7.5	(6.8–8.2)
Medium	7.7	(7.0–8.5)	7.6	(6.9–8.4)	7.6	(6.9–8.4)
High	7.6	(7.0–8.4)	7.5	(6.9–8.4)	7.6	(6.9–8.4)
Very high	7.8	(7.1–8.6)	7.7	(6.9–8.6)	7.7	(7.0–8.6)
**Total**	**7.6**	**(6.9–8.4)**	**7.5**	**(6.8–8.3)**	**7.5**	**(6.9–8.3)**

M = Median

IQR = Interquartile Range

**Table 3:** Proportion of children and adolescents with type 1 diabetes with diabetic ketoacidosis at least once or with severe hypoglycaemia at least once in 2020 by sex, age group and regional socioeconomic deprivation Source: DPV Registry, data status: 15 December 2022; GISD Release 2022 v0.2; own calculations

	Proportion of children and adolescents experiencing diabetic ketoacidosis at least once (n=11,735 girls, n=13,243 boys)						Proportion of children and adolescents experiencing severe hypoglycaemia at least once (n=11,735 girls, n=13,243 boys)					
	Girls		Boys		Total		Girls		Boys		Total	
	%	(95% CI)	%	(95% CI)	%	(95% CI)	%	(95% CI)	%	(95% CI)	%	(95% CI)
**Age (years)**												
<7	2.5	(1.7–3.2)	2.7	(2.0–3.4)	2.6	(2.1–3.1)	3.0	(2.2–3.8)	2.4	(1.8–3.1)	2.7	(2.2–3.2)
7–10	2.5	(2.0–3.0)	2.1	(1.7–2.6)	2.3	(2.0–2.7)	3.2	(2.6–3.7)	3.2	(2.6–3.8)	3.2	(2.8–3.6)
11–13	4.2	(3.6–4.8)	3.1	(2.6–3.6)	3.6	(3.2–4.0)	2.6	(2.1–3.1)	3.4	(2.9–3.9)	3.0	(2.6–3.4)
14–17	4.4	(4.0–4.9)	3.7	(3.3–4.1)	4.0	(3.7–4.4)	3.1	(2.7–3.5)	2.9	(2.5–3.2)	3.0	(2.7–3.2)
**Regional socioeconomic deprivation**												
Very low	2.7	(2.2–3.2)	2.2	(1.7–2.6)	2.4	(2.1–2.7)	2.9	(2.3–3.4)	4.1	(3.5–4.7)	3.5	(3.1–3.9)
Low	4.1	(3.4–4.7)	2.9	(2.4–3.4)	3.5	(3.0–3.9)	3.6	(3.0–4.2)	2.7	(2.2–3.2)	3.1	(2.7–3.5)
Medium	4.0	(3.4–4.6)	3.5	(3.0–4.1)	3.8	(3.4–4.2)	2.9	(2.4–3.4)	2.7	(2.3–3.2)	2.8	(2.5–3.2)
High	3.7	(3.0–4.4)	3.3	(2.7–3.9)	3.5	(3.1–4.0)	2.4	(1.8–2.9)	2.5	(2.0–3.0)	2.4	(2.1–2.8)
Very high	4.7	(3.8–5.5)	4.3	(3.5–5.0)	4.5	(3.9–5.0)	3.2	(2.5–3.9)	2.8	(2.2–3.5)	3.0	(2.5–3.5)
**Total**	**3.8**	**(3.4–4.1)**	**3.2**	**(2.9–3.5)**	**3.4**	**(3.2–3.7)**	**3.0**	**(2.7–3.3)**	**3.0**	**(2.7–3.3)**	**3.0**	**(2.8–3.2)**

**Annex Figure 1:**
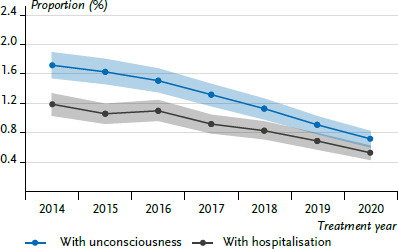
Proportion of children and adolescents with type 1 diabetes experiencing severe hypoglycaemia with unconsciousness or hospitalisation at least once Source: DPV Registry; data status: 15 December 2022

## Appendix

**Annex Table 1:** Relationship between the incidence and prevalence of type 1 diabetes in children and adolescents and regional socioeconomic deprivation in 2020 (T1D incidence n=4,044, T1D prevalence n=32,230); Poisson model adjusted for age, sex, region (North/Central/South) Source: DPV Registry, data status: 03.10.2022; GISD Release 2022 v0.2; own calculations

Incident cases		T1D incidence (95% CI)		Relative risk (95% CI)		p-value
Regional socioeconomic deprivation						
Very low	929	27.3	(25.5–29.0)		1	
Low	854	28.8	(26.8–30.7)	1.028	(0.934–1.130)	0.577
Medium	964	30.8	(28.8–32.7)	1.070	(0.966–1.185)	0.194
High	666	28.2	(26.1–30.4)	0.967	(0.863–1.083)	0.562
Very high	631	31.9	(29.4–34.4)	1.097	(0.977–1.231)	0.116
**Prevalent cases**		**T1D prevalence (95% CI)**		**Relative risk (95% CI)**		**p-value**
**Regional socioeconomic deprivation**						
Very low	7,444	223.4	(218.3–228.5)		1	
Low	6,478	219.5	(214.2–224.9)	0.966	(0.933–0.999)	0.046
Medium	7,460	240.4	(234.9–245.8)	1.036	(0.998–1.074)	0.061
High	5,830	248.7	(242.2–255.1)	1.058	(1.017–1.101)	0.005
Very	5,018	256.2	(249.1–263.3)	1.092	(1.048–1.138)	<0.001

T1D = type 1 diabetes

**Annex Table 2:** Proportion of children and adolescents with type 1 diabetes experiencing severe hypoglycaemia with unconsciousness or hospitalisation at least once in 2020 by sex, age group and regional socioeconomic deprivation Source: DPV Registry, data status: 15 December 2022; GISD Release 2022 v0.2; own calculations

	Proportion of children and adolescents experiencing severe hypoglycaemia with unconsciousness at least once (n=11,735 girls, n=13,243 boys)						Proportion of children and adolescents experiencing severe hypoglycaemia with hospitalisation at least once (n=11,735 girls, n=13,243 boys)					
	Girls		Boys		Total		Girls		Boys		Total	
	%	(95% CI)	%	(95% CI)	%	(95% CI)	%	(95% CI)	%	(95% CI)	%	(95% CI)
**Age (years)**												
<7	0.7	(0.3–1.1)	0.5	(0.2–0.8)	0.6	(0.3–0.8)	0.5	(0.2–0.9)	0.4	(0.1–0.6)	0.4	(0.2–0.6)
7–10	0.6	(0.4–0.9)	0.6	(0.3–0.8)	0.6	(0.4–0.8)	0.5	(0.3–0.7)	0.5	(0.3–0.7)	0.5	(0.3–0.6)
11–13	0.6	(0.4–0.8)	0.9	(0.6–1.1)	0.7	(0.5–0.9)	0.5	(0.3–0.7)	0.7	(0.5–1.0)	0.6	(0.4–0.8)
14–17	0.8	(0.6–1.0)	0.8	(0.6–0.9)	0.8	(0.7–0.9)	0.6	(0.4–0.8)	0.5	(0.4–0.7)	0.6	(0.4–0.7)
**Regional socioeconomic deprivation**												
Very low	0.5	(0.3–0.8)	0.7	(0.5–1.0)	0.6	(0.5–0.8)	0.5	(0.2–0.7)	0.5	(0.3–0.7)	0.5	(0.3–0.7)
Low	0.9	(0.6–1.2)	0.5	(0.3–0.8)	0.7	(0.5–0.9)	0.7	(0.5–1.0)	0.4	(0.2–0.6)	0.6	(0.4–0.7)
Medium	0.8	(0.5–1.1)	0.7	(0.5–1.0)	0.8	(0.6–1.0)	0.5	(0.3–0.7)	0.6	(0.4–0.9)	0.6	(0.4–0.7)
High	0.6	(0.3–0.8)	0.8	(0.5–1.1)	0.7	(0.5–0.9)	0.4	(0.2–0.6)	0.7	(0.4–0.9)	0.5	(0.4–0.7)
Very high	0.7	(0.4–1.1)	0.9	(0.5–1.2)	0.8	(0.6–1.0)	0.6	(0.3–0.9)	0.5	(0.2–0.7)	0.5	(0.3–0.7)
**Total**	**0.7**	**(0.6–0.9)**	**0.7**	**(0.6–0.9)**	**0.7**	**(0.6–0.8)**	**0.5**	**(0.4–0.7)**	**0.5**	**(0.4–0.7)**	**0.5**	**(0.4–0.6)**
