# Corrigendum: Incidence, prevalence and care of type 1 diabetes in children and adolescents in Germany: Time trends and regional socioeconomic situation

**DOI:** 10.25646/11672

**Published:** 2023-08-07

**Authors:** M Buchmann, O Tuncer, M Auzanneau, AJ Eckert, J Rosenbauer

Buchmann M, Tuncer O, Auzanneau M, Eckert AJ, Rosenbauer J et al. (2023)

Journal of Health Monitoring 8(2): 57–78.

DOI 10.25646/11439

In the original version, the source of the GISD data in Figure 1, Table 1, Table 2 and Table 3, Annex Table 1 and Annex Table 2 (‘GISD Release 2022 v0.2’) and information on the availability of the GISD dataset were missing. Reference no. 38 did not cite the most recent paper to the topic.

We added the data source for Figure 1 and the tables, updated the literature, and also included a note on GISD data in the ‘Data availability’ section.

The corrected version of the article is available at DOI 10.25646/11439.2

